# Case Report: Pulmonary enteric adenocarcinoma harboring KRAS/TP53/APC mutations with contralateral lung metastasis: diagnostic challenges and molecular insights

**DOI:** 10.3389/fonc.2025.1726695

**Published:** 2026-01-05

**Authors:** Mengjie Mao, Mengling Hu, Qingxiu Tao, Zhuo Zuo, Bin Liu

**Affiliations:** 1 School of Medical and Life Sciences, Chengdu University of Traditional Chinese Medicine, Chengdu, China; 2 School of Medicine, University of Electronic Science and Technology of China, Chengdu, China; 3 Department of Pathology, Sichuan Cancer Hospital and Institute, Sichuan Cancer Center, Affiliated Cancer Hospital of University of Electronic Science and Technology of China, Chengdu, China; 4 Department of Medical Oncology, Sichuan Cancer Hospital and Institute, Sichuan Cancer Center, Affiliated Cancer Hospital of University of Electronic Science and Technology of China, Chengdu, China

**Keywords:** immunohistochemistry, KRAS, PEAC, synchronous bilateral lung cancer, TP53

## Abstract

Pulmonary enteric adenocarcinoma (PEAC) is a rare subtype of lung adenocarcinoma characterized by intestinal differentiation. We report a case of synchronous bilateral PEAC in an elderly male patient, a presentation that is rarely documented in the literature. Histopathological examination revealed glandular structures resembling colorectal adenocarcinoma, with immunohistochemical positivity for CDX2, CK20, and SATB2, and negativity for TTF-1 and CK7. Molecular testing identified concurrent KRAS, TP53, and APC mutations, suggesting the activation of a colorectal-like oncogenic pathway. Following surgical resection and adjuvant chemoradiotherapy, the patient achieved short-term disease stability. This case highlights the diagnostic dilemma between primary PEAC and metastatic colorectal cancer and underscores the importance of integrated molecular and immunophenotypic profiling for accurate classification and potential targeted therapy.

## Introduction

Pulmonary enteric adenocarcinoma (PEAC) is a rare histologic variant of non-small cell lung cancer (NSCLC) characterized by intestinal differentiation, both morphologically and immunohistochemically. Since its initial description by Tsao and Fraser in 1991, fewer than 200 cases have been reported worldwide. Considering its close resemblance to metastatic colorectal adenocarcinoma (MCRC), distinguishing primary PEAC from MCRC poses a significant diagnostic challenge. While most reported cases involve solitary lesions, bilateral involvement is exceptionally rare (1–3). Moreover, molecular characterization of PEAC remains limited, leaving its pathogenesis and therapeutic vulnerabilities largely unexplored. Here, we present a rare case of bilateral PEAC harboring Kirsten Rat Sarcoma viral oncogene homolog (KRAS), Tumor Protein p53 (TP53), and Adenomatous Polyposis Coli (APC) mutations, and we discuss the diagnostic and molecular implications of these findings.

## Case presentation

A 66-year-old man with a history of type 2 diabetes mellitus and coronary artery disease was incidentally found to have bilateral pulmonary nodules during a routine health check-up. He had a 20-pack-year smoking history (one pack per day for 20 years, quit 8 years ago) and reported no alcohol consumption or chronic pulmonary diseases. His family history was notable for colorectal cancer in both his mother and brother. At presentation, the patient was asymptomatic, with no respiratory or gastrointestinal complaints.

**Figure 1:**
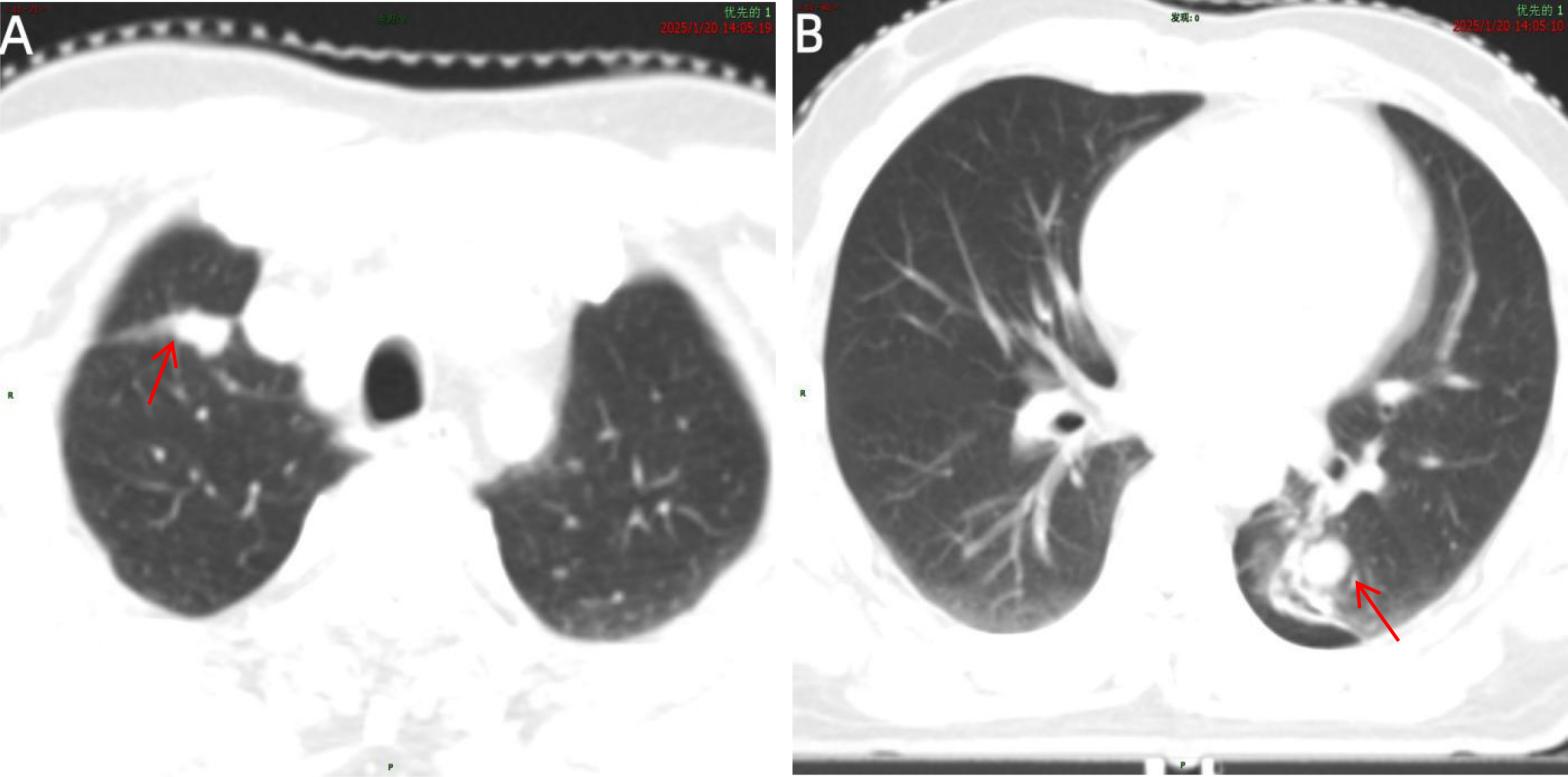
Chest CT (lung window) performed at initial diagnosis. **(A)** A spiculated nodule (arrow) is located at the apex of the right upper lobe. **(B)** A subsolid nodule (arrow) is located in the posterior basal segment of the left lower lobe. Both lesions demonstrate clear margins without mediastinal invasion.

Chest CT revealed a 1.8-cm spiculated solid nodule in the right upper-lobe apex and a 1.2-cm subsolid nodule in the left lower-lobe posterior basal segment. Positron Emission Tomography - Computed Tomography (PET-CT) demonstrated mild fluorodeoxyglucose uptake (SUVmax = 4.2 and 3.8, respectively), with no evidence of mediastinal lymphadenopathy or distant metastasis. **Figure 1** shows the initial preoperative CT examination performed in October 2023. Based on these findings, the clinical stage (cTNM) was determined to be cT1bN0M0 for the right lesion and cT1aN0M0 for the left lesion. Although the patient met the criteria for early-stage disease, a formal multidisciplinary team (MDT) discussion was not initially conducted. This decision was made because of the small size of the bilateral nodules, the absence of mediastinal involvement, and the assumption that the lesions were operable via diagnostic wedge resection.

Subsequently, thoracic surgeons, radiologists, and pathologists collaboratively reviewed the imaging and preliminary findings, leading to the formulation of a treatment plan. We acknowledge that the absence of a formal MDT meeting represents a limitation in this case. Given the bilateral, early-stage nature of the nodules and the patient’s comorbidities, a staged surgical approach was recommended. Bilateral wedge resections were performed via a subxiphoid approach to obtain tissue for diagnosis and molecular profiling. Intraoperative frozen section analysis confirmed adenocarcinoma in both lesions, justifying the limited resection. Systematic lymph node dissection was omitted to preserve lung parenchyma, consistent with the diagnostic intent of the procedure.

During the operation, the right upper-lobe lesion appeared as a firm, tan-white, irregular nodule approximately 1.8 cm in size, located near the apical pleura, with no chest wall invasion or pleural seeding. The left lower-lobe lesion, measuring approximately 1.2 cm, had a partially solid, gelatinous cut surface and was located in the posterior basal segment. No significant pleural adhesions, visceral pleura involvement, effusion, or enlarged hilar/mediastinal lymph nodes were observed. The surrounding lung parenchyma appeared soft, with no emphysematous changes or fibrotic bands.

**Figure 2:**
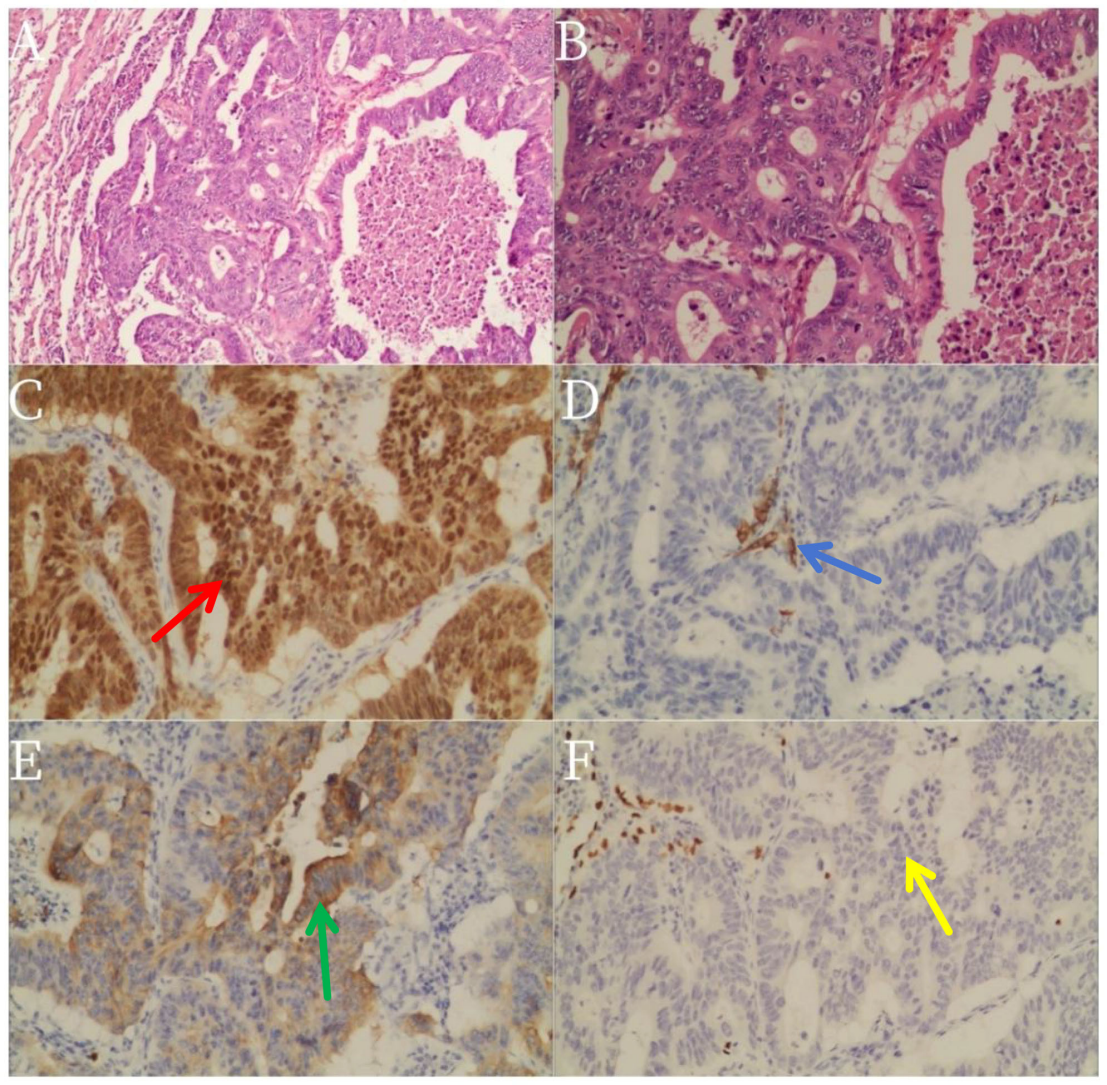
Core biopsies showing the tumor with glandular architecture (H&E stain, magnification **(A)** × 100 and **(B)** × 200). Immunohistochemistry staining pattern of the tumor: colored arrows (red: CDX2; blue: CK7; green: CK20; yellow: TTF-1) indicate the localization of each marker to enhance interpretability. Immunohistochemistry staining pattern of the tumor: **(C)**: CDX **(D)**: CK7 **(E)**: CK20 and **(F)**: TIF-1.

Histopathological examination revealed a moderately differentiated adenocarcinoma with significant necrosis. Immunohistochemistry (IHC) showed negative staining for Cytokeratin 7 (CK7) (−), Thyroid Transcription Factor-1 (TTF-1) (−), and Napsin A (−), while Cytokeratin 20 (CK20) (+), Special AT-rich sequence-Binding protein 2 (SATB2) (+), CDX2 (+), and villin (+) were strongly and diffusely positive (**Figure 2**). Ki-67 expression was 40%–50%. This immunophenotypic pattern, particularly the absence of CK7 and TTF-1, helped distinguish PEAC from other types of lung cancers, especially metastatic adenocarcinomas of the gastrointestinal tract. Both lesions exhibited identical IHC profiles, confirming consistent enteric differentiation.

Molecular profiling identified mutations in KRAS (c.35G>T, p.G12V), TP53 (c.396G>T, p.K132N), and APC (c.3859del, p.I1287*), as well as BRAF amplification (copy number 3.2). Programmed cell Death Ligand-1 (PD-L1) expression was low (TPS < 1%). Comprehensive gastrointestinal evaluation, including gastroscopy and colonoscopy with biopsy, revealed chronic gastritis and several colonic polyps. Colonoscopy identified three polyps (sizes: 0.3–0.8 cm), all histologically consistent with tubular adenomas without dysplasia. There was no evidence of familial adenomatous polyposis (FAP) or germline APC mutation, and no malignancy was detected. These findings supported a primary pulmonary origin for the tumors rather than metastatic colorectal adenocarcinoma. Approximately 14 months following resection, recurrent pulmonary nodules were noted on follow-up CT. PET-CT showed lesions in the right upper-lobe apex and left lower-lobe posterior basal segment, suggesting recurrence, with symmetric, likely reactive mediastinal and hilar lymph nodes. External pathology review confirmed invasive adenocarcinoma with intestinal differentiation. The immunohistochemical profile of the recurrent tumor was consistent with the initial findings, and ALK (D5F3) was negative. Between 15 and 16 months postresection, the patient underwent thoracic radiotherapy (3D-IMRT, 6,000 cGy/15 fractions). At 17–20 months postresection, he received four cycles of pemetrexed (500 mg/m²) and cisplatin (AUC 5–6) chemotherapy, administered every 21 days. Folic acid and vitamin B12 supplementation, as well as dexamethasone premedication, were provided according to standard guidelines. Following this, he received two cycles of maintenance therapy with pemetrexed (500 mg/m²) every 21 days at 21–22 months postresection. Chemotherapy was paused due to treatment-related fatigue and anorexia. Telephone follow-up indicated stable disease.

### Final diagnosis

**Figure 3:**
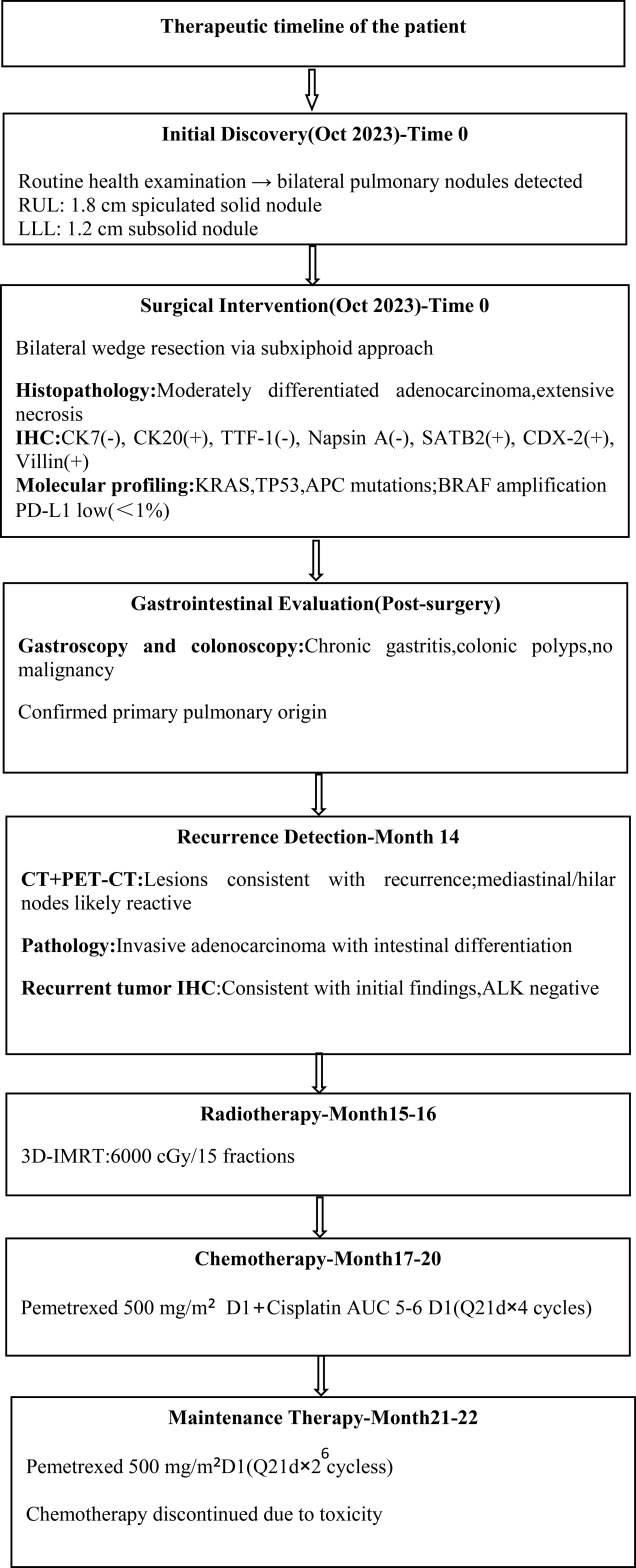
Therapeutic timeline of the patient.

Synchronous bilateral PEAC harboring KRAS, TP53, and APC mutations, with postsurgical recurrence (cT0NxM1, stage IV), is currently stable following radiotherapy and systemic chemotherapy. The patient’s therapeutic timeline is summarized in **Figure 3**.

## Discussion

### Histopathological and clinical characteristics of PEAC

PEAC is a rare histologic variant of NSCLC, exhibiting both morphological and immunohistochemical intestinal differentiation (2). Pathologically, PEAC consists of tall columnar cells arranged in glandular structures that resemble colorectal adenocarcinoma (3). Its exact pathogenesis remains unclear, though some studies suggest a shared embryologic origin between the respiratory and intestinal epithelium. Clinically, PEAC predominantly affects older individuals, with a male predominance, and is often associated with smoking (4). Symptoms are generally nonspecific, including cough, dyspnea, or chest pain, similar to those seen in other forms of lung adenocarcinoma.

Although most reported cases describe solitary lesions, bilateral involvement is extremely rare. This rarity makes the present case, involving synchronous bilateral PEAC, particularly valuable for expanding the clinical understanding and natural history of PEAC. In the current case, both lung lesions exhibited moderately differentiated adenocarcinoma with glandular structures resembling colorectal adenocarcinoma, as well as areas of necrosis.

### Diagnostic challenge: synchronous primaries versus intrapulmonary metastasis

A significant diagnostic challenge in this case was distinguishing synchronous multicentric primary tumors from intrapulmonary metastasis. Molecular analysis provided crucial insights: both lesions exhibited identical and complex KRAS, TP53, and APC mutation profiles (5), strongly supporting a monoclonal origin and favoring a diagnosis of intrapulmonary metastasis over synchronous primary tumors. Initially, the lesions were considered potential synchronous primaries due to their small size, absence of lymph node involvement, and lack of mediastinal spread. Consequently, adjuvant therapy was administered immediately following surgery. However, subsequent molecular profiling revealed identical KRAS, TP53, and APC mutations in both nodules, providing compelling evidence of a shared clonal origin and supporting the diagnosis of intrapulmonary metastasis rather than synchronous primary tumors. This conclusion was drawn after surgery and a comprehensive pathologic review, highlighting the importance of molecular testing for guiding postoperative management. Notably, the identical mutations in TP53 (p.K132N) and APC (p.I1287*) are nonhotspot variants, reinforcing the likelihood of a shared clonal origin rather than independent mutations. This underscores the complexity of diagnosing bilateral PEAC based on clinical and imaging findings alone.

While the molecular analysis strongly supports intrapulmonary metastasis, several clinical and radiologic features initially raised the possibility of synchronous primary tumors (6). These included the absence of mediastinal or hilar lymph node involvement, similar lesion sizes, and negative findings on a comprehensive gastrointestinal evaluation (7). However, these features are not definitive, as intrapulmonary metastasis can occur without lymphatic involvement. Thus, although the possibility of rare multicentric synchronous primaries driven by the same oncogenic pathways cannot be entirely excluded, the molecular evidence strongly favors metastasis from one lesion to another (8).

### Differentiation from metastatic colorectal adenocarcinoma

The differential diagnosis between primary PEAC and MCRC is particularly challenging due to their significant overlap in histomorphology and immunophenotype. This difficulty was further complicated by the patient’s strong family history of colorectal cancer and the tumor’s molecular profile, which included KRAS, TP53, and APC mutations commonly associated with colorectal carcinogenesis (9). To further distinguish PEAC from MCRC, we compared the genomic profiles of PEAC and MCRC using publicly available next-generation sequencing (NGS) datasets. The analysis revealed similarities in key mutations, such as KRAS, TP53, and APC, which are frequently observed in colorectal cancer. Our case findings, including the co-occurrence of KRAS (G12V), TP53 (K132N), and APC (I1287*), are consistent with those described in the literature, suggesting that PEAC may follow a molecular pathogenesis similar to that of MCRC. This molecular concordance strengthens the hypothesis that PEAC shares oncogenic pathways with MCRC, providing insights into its biology and potential therapeutic targets. We acknowledge that even after an exhaustive systemic workup, including PET-CT and pan-endoscopy, the possibility of an occult or regressed primary gastrointestinal lesion cannot be absolutely excluded. It is well-documented that diagnostic modalities have inherent limitations; for instance, colonoscopy can miss small, flat, or intermittently bleeding lesions, and PET-CT may not detect tumors that are small or have low metabolic activity (7). Therefore, the diagnosis of exclusion for PEAC must be approached with rigorous critical appraisal, particularly in a high-risk context like this one. Despite these challenges, the diagnosis of PEAC remains the most compelling conclusion based on the cumulative evidence. Our reasoning is threefold: First, the patient underwent a state-of-the-art, comprehensive search for a primary tumor, which yielded no positive findings (9). Second, while the “colorectal-like” molecular profile is striking, emerging evidence suggests that a subset of PEACs may indeed arise through aberrant activation of the Wnt signaling pathway (Wnt) and Mitogen-Activated Protein Kinase pathway (MAPK) signaling pathways, mimicking colorectal tumorigenesis (10). Recent studies have demonstrated frequent nuclear β-catenin accumulation in PEAC, suggesting aberrant activation of the Wnt/β-catenin pathway similar to colorectal adenocarcinoma (11). Our case provides strong support for this hypothesis. Third, a thorough review of the patient’s prior surgical history (appendectomy and adhesiolysis) revealed no evidence of premalignant or malignant gastrointestinal pathology. Thus, while accepting the theoretical limitations of ruling out MCRC, we assert that PEAC is the most robust and evidence-based diagnosis. This case underscores that, in the context of pulmonary tumors with enteric differentiation, a definitive diagnosis requires a multidisciplinary, integrated approach that critically weighs clinical history, familial risk, comprehensive imaging, endoscopic findings, and deep molecular profiling (12).

### Integrated diagnostic approach: histology, IHC, and molecular profiling

Accurate diagnosis requires the integration of histology, IHC, and molecular testing. The primary challenge is differentiating PEAC from MCRC and conventional lung adenocarcinoma (LUAD) (13–16). Typically, PEAC expresses intestinal markers, including Caudal Type Homeobox 2 (CDX2), CK20, villin, SATB2, and may show variable CK7 or TTF-1 expression (14). MCRC also expresses CDX2 and CK20, but it lacks CK7 and TTF-1. Conversely, LUAD is typically positive for CK7, TTF-1, and Napsin A. Immunohistochemical marker expression in PEAC, MCRC, and LUAD is shown in **Table 1**. Molecularly, KRAS mutations are frequently observed in PEAC, whereas EGFR mutations are rare. MSI and MMR deficiency may occur in a subset of cases, which could have therapeutic implications for immunotherapy (13). This integrated diagnostic approach underscores the importance of combining histopathological, immunophenotypical, and molecular data to resolve complex clinical dilemmas, particularly in patients with a family history of colorectal cancer (15). CK7 expression in PEAC is variable: most cases show complete loss of CK7 expression (CK7−), while a minority may exhibit focal or weak positivity (CK7±).

**Table 1:** Immunohistochemical marker expression in PEAC, MCRC, and LUAD.

Marker	PEAC	MCRC	LUAD
CK7	±/− (variable or absent)	−	+
CK20	+	+	−
CDX2	+	+	−
SATB2	+	+	−
TTF-1	−	−	+
Napsin A	−	−	+
APC mutation	Occasionally mutated	Frequently mutated	Rare
TP53 mutation	Common mutations	Common mutations	Common mutations
KRAS mutation	G12V	Common mutations	Rare

**Figure 4:**
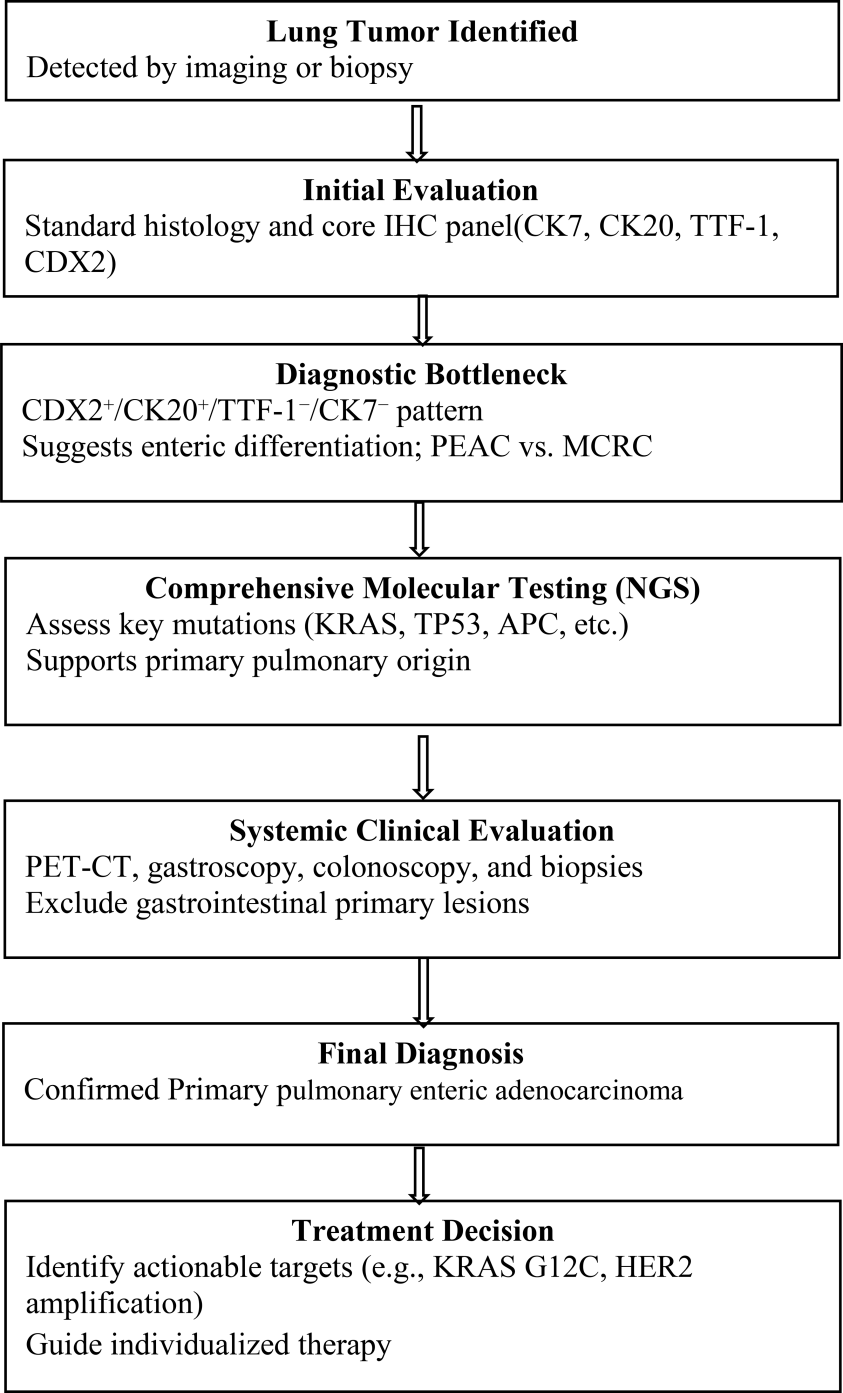
Diagnostic workflow for suspected PEAC.

This case highlights the critical importance of an integrated diagnostic workflow for tumors with overlapping morphological and immunophenotypic features. Standard histology and a core IHC panel (CK7, CK20, TTF-1, CDX2, etc.) can suggest enteric differentiation, as in CDX2+/CK20+/TTF-1−/CK7− tumors, but cannot reliably distinguish PEAC from MCRC. At this diagnostic bottleneck, comprehensive NGS serves as the decisive tool. In our patient, co-occurring KRAS, TP53, and APC mutations—a classic colorectal-like molecular signature—provided critical insight into tumor biology, shifting the focus from an ambiguous immunophenotype to defined molecular pathways. After a thorough clinical evaluation excluded a gastrointestinal primary, this evidence enabled confident classification as primary PEAC. The true strength of this workflow lies not in novelty but in its structured, timely integration of molecular profiling, ensuring accurate identification of rare entities, minimizing misdiagnosis, and revealing actionable targets such as KRAS G12C or HER2 amplification to guide precision therapy. The diagnostic workflow for suspected PEAC is shown in **Figure 4**. The diagnostic algorithm summarized in **Figure 4** is consistent with the current evidence base for PEAC and rare-variant lung adenocarcinomas, even though no formal PEAC-specific guideline exists. Published clinicopathological and genomic studies support the use of a stepwise workflow incorporating morphology, immunohistochemistry (IHC), and molecular profiling. Multiple PEAC series have emphasized the diagnostic value of intestinal differentiation markers (CDX2, CK20, villin) together with loss of lung-lineage markers (CK7, TTF-1), especially in distinguishing PEAC from MCRC (11). Genomic studies further support this algorithm: in a 16-case cohort, KRAS and TP53 mutations were among the most frequent alterations (16); a larger 24-case next-generation sequencing study confirmed TP53 (70.6%) and KRAS (47.1%) as major drivers and highlighted the molecular heterogeneity of PEAC (17). Importantly, whole-exome sequencing combined with DNA methylation profiling has demonstrated that PEAC possesses distinct molecular features compared with lung metastatic colorectal cancer, reinforcing the necessity of combining IHC with molecular testing during diagnostic evaluation (4). Case series and clinical reviews similarly recommend comprehensive systemic evaluation—including PET-CT, colonoscopy, and gastroscopy—to exclude an occult gastrointestinal primary before confirming PEAC (18). Therefore, although **Figure 4** is not based on formal guidelines, each step is grounded in reproducible evidence from molecular pathology, thoracic oncology, and published PEAC case series, reflecting a rational, evidence-supported diagnostic approach.

### Treatment experience and literature review

Previous studies have shown that PEAC tends to exhibit aggressive biological behavior with early metastatic potential. Currently, no standardized treatment strategy for PEAC exists, and most patients are managed according to regimens used for conventional lung adenocarcinoma. Surgical resection remains the mainstay of therapy for early-stage disease, usually followed by adjuvant treatment. In contrast, advanced-stage patients are treated with chemotherapy, radiotherapy, targeted therapy, or immunotherapy (12). According to the literature, among 28 reported PEAC patients (3), only five died within 1–20 months after surgery, suggesting a potentially favorable outcome when complete resection is achieved. In the present case, tumor recurrence was observed 15 months after the initial surgery, which was not followed by adjuvant therapy. Chemotherapy is the most commonly used adjuvant modality for PEAC. Some studies have reported that regimens containing pemetrexed or paclitaxel plus platinum are effective, while XELOX or FOLFIRI schemes show poor response (19). Although carboplatin combined with paclitaxel is the most widely used regimen, the only documented successful case achieved disease control after four cycles of pemetrexed and carboplatin. Qureshi et al. reported one patient who achieved disease stability following four cycles of pemetrexed plus carboplatin (22). Teranishi et al. described a stage IVB PEAC patient harboring a KRAS G12D mutation and PD-L1 TPS < 1%, who received palliative radiotherapy followed by combination therapy with pembrolizumab, pemetrexed, and carboplatin, resulting in a partial response (PR) (20). Todisco et al. documented a case of PEAC with multiple metastases that responded to systemic therapy consisting of pemetrexed, cisplatin, and bisphosphonates, with significant relief of bone pain after pelvic radiotherapy (20 Gy in five fractions). In our case, the patient received four cycles of cisplatin plus pemetrexed followed by two cycles of maintenance chemotherapy, and the disease has remained stable to date. A summary of reported PEAC cases, including treatment modalities and outcomes, is provided in **Table 2**.

**Table 2:** Summary of published cases of pulmonary enteric.

Reference	No. of cases/Stage	Key molecular findings	Treatment	Response	Survival
Tu et al. (18)	6 cases (early and advanced)	Not specified	Surgical resection ± adjuvant chemotherapy	Variable; recurrence in 2 patients	5/6 alive at last follow-up (median OS not reported)
Kraus et al. (10)	Review/single-center series	Low TTF-1, enteric differentiation	Surgery + systemic therapy (chemotherapy/radiotherapy)	Limited response to systemic therapy	Generally poor prognosis
Guo et al. (19)	1 (case)	Not reported	1L pemetrexed/platinum → 2L FOLFIRI (XELOX/FOLFIRI ineffective)	Progressive disease after 2L	Survival < 20 months
Qureshi et al. (year not specified)	1 (case)	Not reported	Pemetrexed + carboplatin × 4 cycles; stable disease	Stable disease	Ongoing follow-up
Teranishi et al. (20)	1 (stage IVB)	KRAS G12D; PD-L1 TPS < 1%	Radiotherapy → pembrolizumab + pemetrexed + parboplatin	Partial response (PR)	Alive at last follow-up
Todisco et al. (year not specified)	1 (case)	Not reported	Pemetrexed + cisplatin + bisphosphonate; 20 Gy pelvic RT	Symptomatic relief; radiologic improvement	Follow-up duration not stated
Present case (Authors, 2025)	1 (stage IV, recurrent)	KRAS G12D; TP53 p.K132N, APC p.l1287*	Cisplatin + pemetrexed × 4 → maintenance pemetrexed × 2 cycles → radiotherapy	Stable disease > 20 months postsurgery	Alive (stable disease)
Kishikawa et al. (11)	5 (per WHO criteria)	Intestinal markers (CDX2, CK20, HNF4α, MUC2, SATB2); nuclear β-catenin in 3/5; APC and TP53 mutations; no EGFR/ALK alterations	Not specified	–	–

### Molecular pathogenesis and therapeutic implications

Comprehensive genomic profiling revealed concurrent KRAS, TP53, and APC mutations in this patient—an exceptionally rare combination in PEAC. These alterations are canonical drivers of colorectal carcinogenesis and suggest that PEAC may arise through an intestinal-type oncogenic cascade involving aberrant activation of the Wnt/β-catenin and MAPK pathways (21). Although publicly available NGS datasets for PEAC are limited, we conducted a comparative review of published genomic studies, including datasets from Zuo et al., Jurmeister et al., and Kishikawa et al. These reports collectively show that PEAC frequently carries KRAS mutations and may harbor occasional APC or TP53 alterations, partially overlapping with the classical colorectal adenocarcinoma mutation spectrum. The co-occurrence of KRAS, TP53, and APC mutations in our patient mirrors the colorectal-like molecular signature described in these studies, further supporting the hypothesis that PEAC follows a similar Wnt/MAPK-driven oncogenic pathway (5, 7, 14).

From a therapeutic perspective, these findings highlight the potential for targeted precision therapies. This molecular signature provides a plausible explanation for the tumor’s intestinal differentiation and aggressive biological behavior, supporting the hypothesis that PEAC represents a molecularly distinct colorectal-like variant of lung adenocarcinoma (22, 23). From a therapeutic perspective, these findings highlight the potential of targeted precision approaches. Agents directed at KRAS (e.g., sotorasib or adagrasib) or modulating the Wnt/β-catenin axis, as well as HER2-targeted therapies in selected patients, may offer future options (23). However, given the absence of standardized protocols and the frequent occurrence of early metastasis, clinical outcomes for PEAC remain unsatisfactory, emphasizing the need for further molecularly guided trials (24).

### Role of immunotherapy and biomarker testing

Deficiency in mismatch repair (dMMR) and microsatellite instability-high (MSI-H) is emerging as a predictive biomarker for immune checkpoint inhibitor (ICI) efficacy across multiple tumor types. Loss of MMR function leads to the accumulation of insertion/deletion mutations and frameshift neoantigens, thereby enhancing tumor immunogenicity and sensitivity to PD-1/PD-L1 blockade. Although MSI-H/dMMR status has been extensively studied in colorectal and endometrial cancers, its prevalence and clinical significance in PEAC remain largely unexplored because of the rarity of this entity (25).

Several recent reports have identified MSI-H or MMR-deficient PEACs that showed objective responses or durable disease control after ICI therapy, suggesting that PEAC may include an immune-responsive molecular subset. These findings underscore the molecular heterogeneity of PEAC and its potential sensitivity to immunotherapy in certain patients (26). In the present case, MSI/MMR testing could not be performed due to limited residual tissue after molecular analyses, which is an important limitation we acknowledge. Determining the MSI/MMR status might have provided additional guidance for treatment selection, as MSI-H tumors could respond to immunotherapy (e.g., pembrolizumab), whereas microsatellite-stable tumors would likely favor chemotherapy or targeted therapy (27).

Therefore, we emphasize that sufficient tissue sampling should always be obtained for suspected PEAC, not only for molecular profiling but also for key biomarkers such as MSI/MMR, PD-L1, and tumor mutational burden (TMB), which inform personalized treatment decisions (28).

## Conclusion

This case report describes a rare instance of synchronous bilateral PEAC characterized by a triple mutation of KRAS, TP53, and APC, a molecular signature closely resembling the classic oncogenic pathway observed in colorectal cancer. This case highlights the significant diagnostic challenge of distinguishing primary PEAC from MCRC, given their substantial overlap in histomorphology and immunophenotype. Our experience confirms that IHC alone is insufficient for establishing a definitive diagnosis.

Therefore, we implemented an integrated diagnostic workflow that combines histological evaluation, IHC analysis (including CK7, CK20, CDX2, and TTF-1), comprehensive NGS, and detailed clinical and endoscopic examinations. This approach successfully ruled out a gastrointestinal primary tumor and established the diagnosis of PEAC. The “colorectal-like” molecular profile (coexistence of KRAS, TP53, and APC mutations) in this case provides strong evidence supporting the hypothesis of “enteric-type” carcinogenesis in PEAC and highlights the critical roles of the Wnt and MAPK signaling pathways.

Although the patient’s condition has remained stable following surgery, radiotherapy, and pemetrexed-based chemotherapy, treatment for PEAC remains nonstandardized, underscoring the urgent need to improve clinical outcomes.

## Future perspectives

Future research and clinical practice for PEAC should focus on the following key directions:

1. Establishing standardized treatment guidelines: Due to the extreme rarity of PEAC, no established treatment standards currently exist, and patients are typically managed according to protocols for conventional lung adenocarcinoma. Future efforts should involve multicenter collaborations and case registries to accumulate clinical data, enabling identification of the most effective chemotherapy regimens, targeted therapies, and immunotherapy strategies for PEAC.
2. Deepening molecular subtyping for precision medicine: The “colorectal-like” molecular signature observed in this case suggests that PEAC may comprise distinct molecular subtypes. Future studies should systematically characterize the molecular landscape of PEAC to uncover additional therapeutic targets. Targeted agents against KRAS (e.g., KRAS G12C inhibitors), HER2 amplification, and the Wnt/β-catenin pathway may provide new therapeutic options for select patient populations.
3. Emphasizing the need for immunotherapy biomarker testing: dMMR or MSI-H is a robust predictive biomarker for the efficacy of immune checkpoint inhibitors. Given reports that MSI-H PEAC patients can benefit from immunotherapy, MSI/MMR testing should be integrated as a routine component of the diagnostic workflow. This will help identify patients sensitive to PD-1/PD-L1 inhibitors and enable personalized immunotherapy.
4. Promoting the application of an optimized diagnostic workflow: The integrated diagnostic workflow we proposed—combining histology, IHC, NGS, and clinical evaluation—is crucial for accurately differentiating PEAC from MCRC. Standardization and widespread adoption of this workflow should be promoted to reduce misdiagnosis and ensure that comprehensive information, including actionable mutations and immune biomarkers, is available at diagnosis to guide optimal, personalized treatment plans.

## Data Availability

The original contributions presented in the study are included in the article/**Supplementary Material**. Further inquiries can be directed to the corresponding author.
