# Supplementary material for: Case Report: Pulmonary enteric adenocarcinoma harboring KRAS/TP53/APC mutations with contralateral lung metastasis: diagnostic challenges and molecular insights
Source: Front Oncol. 2026 Jan 5;15:1726695. doi: 10.3389/fonc.2025.1726695 (PMC12812567; doi:10.3389/fonc.2025.1726695)
Supplement: Supplementary file 1 [file DataSheet1.docx]

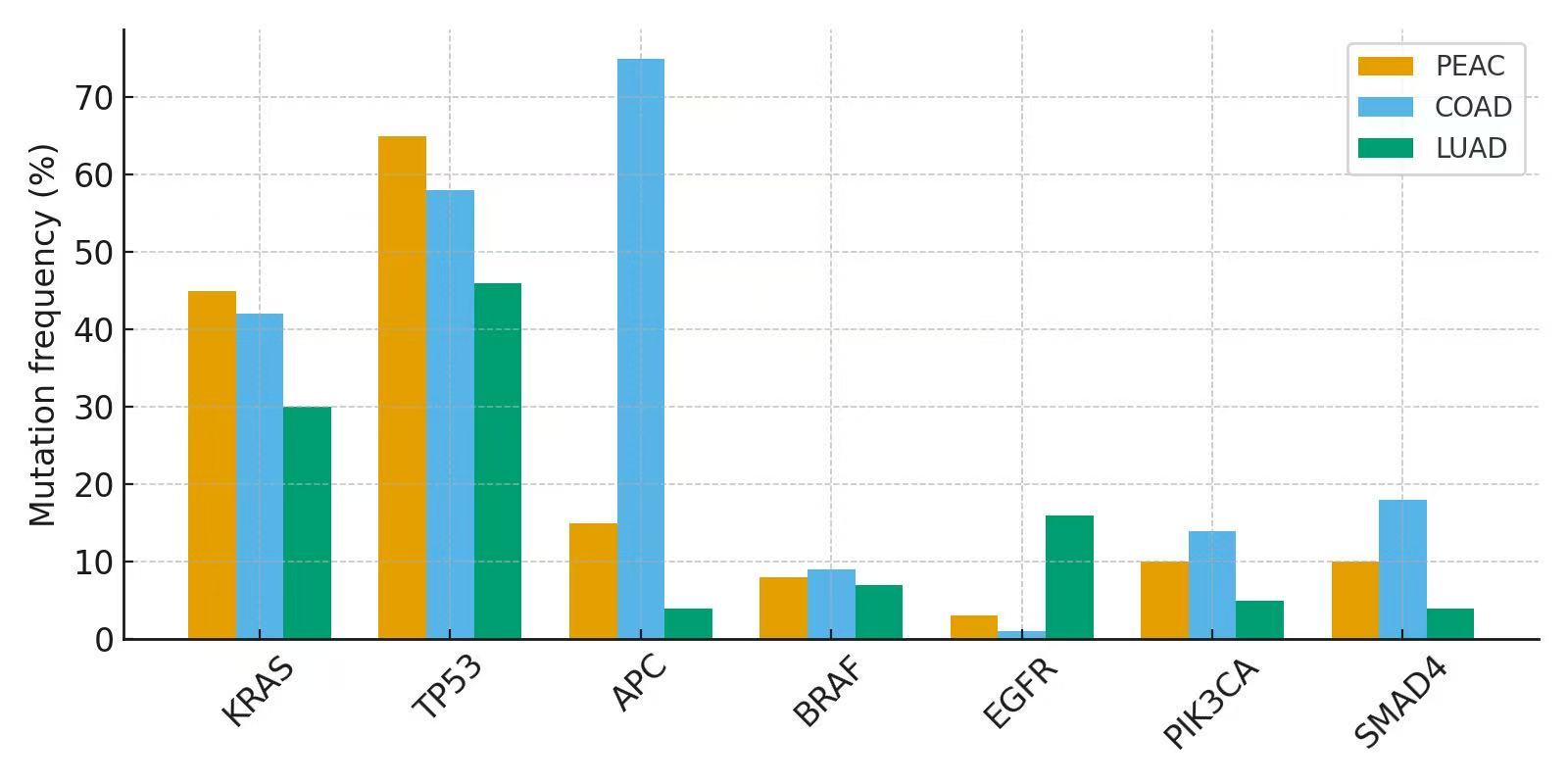


Figure S1:presents a comparison of mutation frequencies in KRAS, TP53, and APC across PEAC, Colorectal Adenocarcinoma(COAD), and lung adenocarcinoma (LUAD). The chart shows that PEAC exhibits mutation frequencies in KRAS and TP53 that are similar to those seen in COAD, while APC mutations are notably more frequent in PEAC compared to both LUAD and COAD. This comparison further supports the hypothesis that PEAC may share molecular features with colorectal cancer.


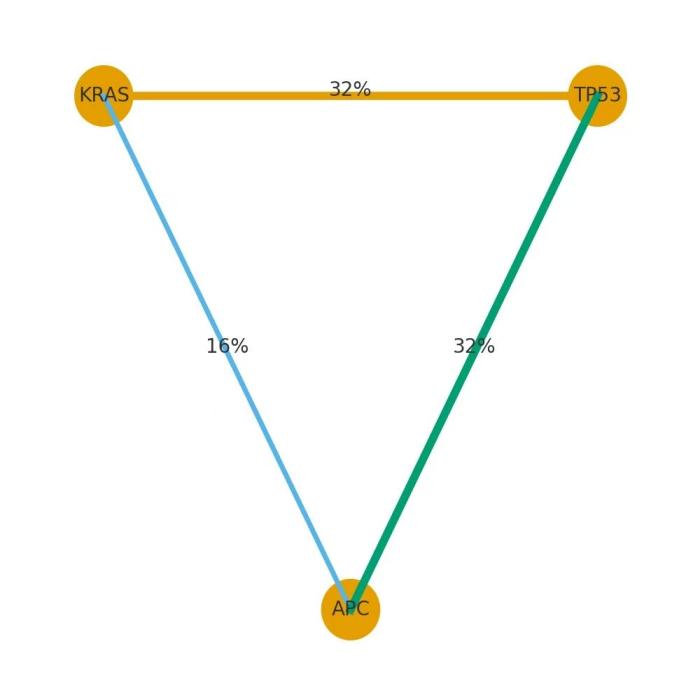


Figure S2:illustrates the co-occurrence of KRAS, TP53, and APC mutations in PEAC. The diagram highlights that 32% of cases exhibit co-mutation between KRAS and TP53, 16% between KRAS and APC, and 32% between TP53 and APC. This visualization provides an intuitive understanding of the co-existing mutation patterns in PEAC, supporting the notion that PEAC may follow a molecular pathway similar to that of colorectal cancer.
